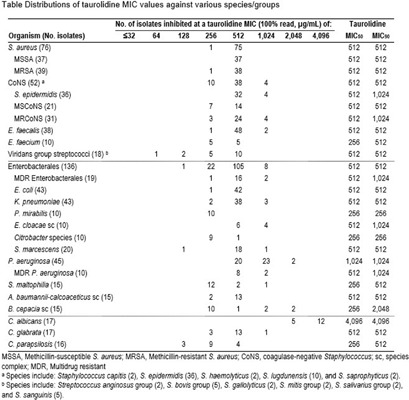# In Vitro Antimicrobial Activity of Taurolidine against Isolates Associated with Catheter-Related Bloodstream Infections

**DOI:** 10.1017/ash.2024.267

**Published:** 2024-09-16

**Authors:** Jared Crandon, S.J. Ryan Arends, Paul Rhomberg, Mariana Castanheira

**Affiliations:** Cormedix Inc.; JMI Labs / Element; Element Iowa City

## Abstract

**Background:** Taurolidine exhibits broad antimicrobial activity and is a component of a recently FDA approved catheter lock solution (DefenCath®, taurolidine 13,500 μg/mL and heparin 1000 Units/mL) indicated for reducing the risk of catheter-related bloodstream infections (CRBSI) in adult patients receiving chronic hemodialysis through a central venous catheter (HD-CVC). FDA approval was based on a Phase 3 randomized trial (LOCK-IT-100) in which DefenCath showed a 71% reduction in CRBSI risk among HD-CVC patients as compared with heparin alone. Although individual isolates from the clinical program were not available for testing, this study evaluated the in vitro antimicrobial activity of taurolidine against a set of recent clinical isolates representative of those recovered from the LOCK-IT-100 trial and/or those commonly associated with CRBSI. **Methods:** 420 bacterial and 50 yeast isolates were selected from the SENTRY Antimicrobial Surveillance Program. All isolates were collected from the bloodstream of patients in the U.S. between 2018-2023. Isolates were tested for susceptibility to taurolidine and comparators using Clinical and Laboratory Standards Institute (CLSI) broth microdilution guidelines. JMI Laboratories produced susceptibility test panels for testing. CLSI-recommended quality control strains were also tested concurrently. MIC values were determined after 24 hours. **Results:** Taurolidine exhibited broad antimicrobial activity against all isolates tested (see table). Against gram-positive bacteria, taurolidine MIC50/90 values ranged from 256-512/512-1,024 μg/mL for S. aureus, Coagulase-negative Staphylococcus, Enterococcus species, and Viridans group streptococci. This activity was maintained regardless of methicillin susceptibility for Staphylococcal isolates or vancomycin resistance among Enterococcal species. Against gram-negative bacteria, taurolidine MIC50/90 values ranged from 256-1,024/512-2,048 μg/mL for Enterobacterales, P. aeruginosa, S. maltophilia, A. baumannii-calcoaceticus, and B. cepacia. This activity was maintained in both multidrug resistant Enterobacterales and P. aeruginosa isolates. Among Candida isolates, taurolidine MIC50/90 values ranged from 256-512/512 μg/mL for C. glabrata and C. parapsilosis while taurolidine MIC50/90 values of 4,096/4,096 μg/mL were observed for C. albicans. **Conclusions:** Taurolidine activity was very similar among a large collection of gram-positive, gram-negative, and yeast organisms. MIC90 values for all species/groups were ≤2,048 μg/mL, except C. albicans where an MIC90 of 4,096 μg/mL was observed. The activity of taurolidine was unaffected by resistance to antibiotics (i.e. methicillin, vancomycin, or multi-drug resistance) among gram-positive or gram-negative organisms. Based on these data, catheter lock solutions containing the broad-spectrum antimicrobial taurolidine at 13,500 μg/mL have the potential to prevent CRBSI caused by a variety of species, including those observed in the recent LOCK-IT-100 clinical trial and other common bloodstream pathogens

**Figure:**